# Supplementary material for: Tissue-specific regulation of BMP signaling by Drosophila N-glycanase 1
Source: eLife. 2017 Aug 4;6:e27612. doi: 10.7554/eLife.27612 (PMC5599231; doi:10.7554/eLife.27612)
Supplement: Transparent reporting form [file elife-27612-transrepform.docx]

***eLife’s* transparent reporting form**

We encourage authors to provide detailed information *within their submission* to facilitate the interpretation and replication of experiments. If you have any questions, please contact us: [editorial@elifesciences.org](mailto:editorial@elifesciences.org).

**Sample-size estimation**

- You should state whether an appropriate sample size was computed when the study was being designed
- You should state the statistical method of sample size computation and any required assumptions
- If no explicit power analysis was used, you should describe how you decided what sample (replicate) size (number) to use

Please outline where this information can be found within the submission (e.g., page numbers or figure legends), or explain why this information doesn’t apply to your submission:

We did not calculate sample sizes for this study. A large number of animals (80-200) was scored for each genotype in rescue studies except for experiments involving adult *Pngl*-null escapers (Figure 1C and 1D), in which only 20 animals were used per genotype due to the scarcity of these animals. All numbers are shown in the Figures.

**Replicates**

- You should report how often each experiment was performed
- You should include a definition of biological versus technical replication
- The data obtained should be provided and sufficient information should be provided to indicate the number of independent biological and/or technical replicates
- If you encountered any outliers, you should describe how these were handled
- Criteria for exclusion/inclusion of data should be clearly stated
- High-throughput sequence data should be uploaded before submission, with a private link for reviewers provided (these are available from both GEO and ArrayExpress)

Please outline where this information can be found within the submission (e.g., page numbers or figure legends), or explain why this information doesn’t apply to your submission:

The Western blots were performed at least three times independently (mentioned either in the figure legend (Figure 1G) or in the methods section (Figure 3O, Figure 7J and 7K, and Figure 7–figure supplement 3)). Due to the small number of escapers obtained and used in Figure 1C and D, these experiments were repeated three independent times (mentioned in the figure legend). All phenotypes shown in the manuscript have been analyzed in multiple animals from at least two independent crosses.

**Statistical reporting**

- Statistical analysis methods should be described and justified
- Raw data should be presented in figures whenever informative to do so (typically when N per group is less than 10)
- For each experiment, you should identify the statistical tests used, exact values of N, definitions of center, methods of multiple test correction, and dispersion and precision measures (e.g., mean, median, SD, SEM, confidence intervals; and, for the major substantive results, a measure of effect size (e.g., Pearson's r, Cohen's d)
- Report exact p-values wherever possible alongside the summary statistics and 95% confidence intervals. These should be reported for all key questions and not only when the p-value is less than 0.05.

We have not used statistical analysis in our study, as the majority of the phenotypes that we report seem to be all-or-none.

Please outline where this information can be found within the submission (e.g., page numbers or figure legends), or explain why this information doesn’t apply to your submission:

(For large datasets, or papers with a very large number of statistical tests, you may upload a single table file with tests, Ns, etc., with reference to page numbers in the manuscript.)

**Additional data files (“source data”)**

- We encourage you to upload relevant additional data files, such as numerical data that are represented as a graph in a figure, or as a summary table
- Where provided, these should be in the most useful format, and they can be uploaded as “Source data” files linked to a main figure or table
- Include model definition files including the full list of parameters used
- Include code used for data analysis (e.g., R, MatLab)
- Avoid stating that data files are “available upon request”

Please indicate the figures or tables for which source data files have been provided:

None
